# In Silico and Glioblastoma Cell Line Evaluation of Thioflavin-Derived Zinc Nanoparticles Targeting Beclin Protein

**DOI:** 10.7759/cureus.69319

**Published:** 2024-09-13

**Authors:** Parineeta Dandagi, Yuvaraj Babu K, Taniya Mary Martin, Meenakshi Sundaram K

**Affiliations:** 1 Department of Anatomy, Saveetha Dental College and Hospitals, Saveetha Institute of Medical and Technical Sciences, Saveetha University, Chennai, IND

**Keywords:** autophagy regulation, beclin protein, cytotoxic effects, glioblastoma, molecular docking, nanotechnology, thioflavin-derived zinc nanoparticles

## Abstract

Introduction: This study explores the anticancer potential of Thioflavin-derived zinc nanoparticles (Th-ZnNPs) using both in vitro and in silico methods. Thioflavin, known for its specific binding properties, faces challenges such as bioavailability, rapid metabolism, and solubility. To overcome these limitations and enhance therapeutic efficacy, nanotechnology was utilized to synthesize Th-ZnNPs. These nanoparticles (NPs) are designed to improve drug delivery and effectiveness. The Beclin protein, which plays a critical role in regulating autophagy in cancer cells, was identified as a potential target for these NPs. The study aims to evaluate the interaction between Th-ZnNPs and Beclin protein in glioblastoma cell lines and assess the potential of these NPs as novel anticancer agents.

Methods: Th-ZnNPs were synthesized using advanced nanotechnology techniques to improve the bioavailability and solubility of Thioflavin. To explore their anticancer potential, in silico analyses were performed, including molecular docking studies to evaluate the binding affinity between the ZnNPs and Beclin protein, which is integral to autophagy regulation. This computational approach identified the Beclin protein as a promising target for the ZnNPs. Complementary in vitro assays were then conducted, where glioblastoma cell lines (procured from the National Centre for Cell Science, Pune, India) were treated with ZnNPs to assess their cytotoxic effects. The assays also included mechanistic studies to validate the interaction between ZnNPs and Beclin protein and to understand their influence on autophagy pathways.

Results: The synthesis of Th-ZnNPs successfully enhanced their solubility and bioavailability compared to Thioflavin alone. In silico findings showed a strong binding affinity between the Th-ZnNPs and the Beclin protein, suggesting that these NPs may effectively target cancer cells through this interaction. Beclin protein was validated as a relevant target due to its critical role in autophagy regulation. In vitro assays further confirmed the anticancer potential of the Th-ZnNPs, as they exhibited significant cytotoxic effects on glioblastoma cells. Additionally, mechanistic studies revealed that Th-ZnNPs impact Beclin protein and modulate autophagy pathways, supporting their proposed role as effective anticancer agents.

Conclusions: The study highlights the promising anticancer potential of Th-ZnNPs. By overcoming the limitations of Thioflavin through nanotechnology, these NPs show significant therapeutic promise in targeting glioblastoma cells. The strong binding affinity between Th-ZnNPs and the Beclin protein, coupled with confirmed cytotoxic effects, underscores their potential as novel anticancer agents. This integrated approach not only enhances the delivery and efficacy of Thioflavin but also opens new avenues for targeted therapy in glioblastoma treatment.

## Introduction

Glioblastoma multiforme (GBM) is the most aggressive and prevalent brain tumor in adults, distinguished by its rapid growth and extensive infiltration into surrounding brain tissue [1,2]. Despite advancements in surgical techniques, radiation therapy, and chemotherapy, the prognosis for GBM remains grim, with a median survival time of approximately 15 months post-diagnosis. The poor prognosis is largely due to GBM's inherent resistance to conventional therapies and its ability to infiltrate and evade treatment, underscoring the need for innovative therapeutic strategies [3-5]. Nanotechnology has emerged as a transformative approach in the quest to improve cancer treatment outcomes. By manipulating materials at the molecular scale, nanotechnology enables the development of nanoparticles (NPs) with unique properties that enhance drug delivery and targeting. These properties include a high surface area to volume ratio, tunable size, and the ability to be functionalized with specific specific targeting ligands. Among various NPs, zinc nanoparticles (ZnNPs) have attracted significant attention due to their biocompatibility and potential therapeutic benefits. ZnNPs can be engineered to optimize their size and surface characteristics, making them suitable for a range of biomedical applications, including drug delivery and diagnostic imaging [6-8]. A particularly promising strategy involves conjugating ZnNPs with Thioflavin, a benzothiazole dye known for its ability to bind specifically to certain protein structures [9-12]. Thioflavin is traditionally used to detect amyloid fibrils in neurodegenerative diseases, but its binding properties can also be harnessed for targeted cancer therapies. By attaching Thioflavin to ZnNPs, it is possible to enhance the specificity of drug delivery systems, improving the efficacy of therapeutic agents while minimizing off-target effects.

One key target in this context is the Beclin protein, a critical regulator of autophagy, the cellular process involved in the degradation and recycling of cellular components. Autophagy plays a dual role in cancer: it can suppress tumorigenesis by eliminating damaged cells, but it can also enable cancer cells to survive under stress and treatment conditions. In GBM, Beclin's role in autophagy presents a novel therapeutic target [2-4]. Modulating Beclin activity with ZnNPs may influence the autophagic response in cancer cells, potentially leading to enhanced cell death and reduced tumor growth. Molecular docking studies have demonstrated that ZnNPs exhibit a strong binding affinity for the Beclin protein. This interaction suggests that these NPs could effectively disrupt the autophagy process in GBM cells, promoting cell death and potentially overcoming some of the limitations of conventional therapies [13]. However, these computational predictions must be validated through in vitro assays. In vitro experiments using glioblastoma cells, such as the human glioblastoma cell line (U87) and malignant glioblastoma tumor cell line (U251), are essential for assessing the cytotoxic effects of thioflavin-derived ZnNPs (Th-ZnNPs) [14]. These assays will evaluate the impact of these NPs on cell viability, apoptosis induction, and cell proliferation, providing empirical evidence of their therapeutic potential. The results will help determine optimal NP concentrations and exposure times, guiding future therapeutic strategies [5-9].

## Materials and methods

This study employed a combination of in vitro and in silico techniques to investigate the anticancer potential of Th-ZnNPs and their interaction with Beclin protein, focusing on glioblastoma cell line evaluation. The methodology included the synthesis and characterization of the NPs, biological assays to evaluate anticancer activity, and computational modeling to explore potential interactions between Thioflavin and Beclin protein.

Synthesis of Th-ZnNPs

All of the chemicals used in this section were analytical grade and procured from HiMedia, Mumbai, India. Th-ZnNPs were synthesized using a sol-gel method. Zinc acetate dihydrate (Zn(CH₃COO)₂·2H₂O) was used as the zinc precursor, and Thioflavin was incorporated as the stabilizing and functionalizing agent. Briefly, Zn(CH₃COO)₂·2H₂O was dissolved in ethanol and mixed with a Thioflavin solution prepared in dimethyl sulfoxide (DMSO). The mixture was stirred at room temperature for two hours to ensure complete reaction. The resulting solution was then aged for 24 hours, followed by the addition of deionized water to precipitate the NPs. The precipitate was collected, washed with ethanol, and dried at 60°C for 12 hours [7,8].

Characterization of Th-ZnNPs

The Th-ZnNPs were characterized using Fourier-transform infrared spectroscopy (FTIR), ultraviolet-visible (UV-Vis) spectroscopy, and X-ray diffraction (XRD). FTIR spectra were recorded using a PerkinElmer FTIR spectrometer (PerkinElmer, Waltham, USA) in the range of 4000 to 400 cm⁻¹ to identify functional groups and confirm the presence of Thioflavin on the ZnNPs. UV-Vis spectra were obtained using a Shimadzu UV-1800 spectrophotometer (Shimadzu, Kyoto, Japan), and the absorbance was measured in the range of 200 to 800 nm to analyze the optical properties of the NPs. XRD analysis was performed with a Rigaku X-ray diffractometer (Rigaku, Tokyo, Japan), using Cu Kα radiation (λ = 1.5406 Å) to determine the crystalline structure and phase purity of the ZnNPs [1-5].

Cell culture and treatment

For the study, glioblastoma cell lines (procured from the National Centre for Cell Science, Pune, India) were selected and cultured in appropriate media supplemented with fetal bovine serum (Gibco, India) and antibiotics (pen strep, Gibco) under standard conditions (37°C, 5% CO_2_). To mimic glioblastoma conditions, cells were treated with Th-ZnNPs at varying concentrations following the induction of inflammatory responses. Experimental groups included a control group, an inflammation-induced group, and groups treated with Th-ZnNPs at different concentrations. This approach aimed to evaluate the efficacy of Th-ZnNPs as potential anticancer agents by assessing their impact on glioblastoma cell viability and inflammation markers [9,10].

Cell viability assay

To assess glioblastoma cell viability, the 3-(4,5-dimethylthiazol-2-yl)-2,5-diphenyltetrazolium bromide (MTT) (HiMedia, India) assay was conducted. Glioblastoma cells were seeded in 96-well plates at a density of 5 × 10⁴ cells/well and exposed to various concentrations of Th-ZnNPs for 48 hours. Following treatment, 20 µL of MTT solution (5mg/mL in phosphate-buffered saline) was added to each well and incubated for four hours. Formazan crystals formed were dissolved in DMSO (HiMedia, India), and absorbance was measured at 570 nm using a microplate reader. Cell viability percentages were calculated relative to untreated controls. The statistical significance was analyzed using a two-way analysis of variance (ANOVA) and the Bonferroni post hoc test [11].

Gene expression analysis

This study focused on gene expression analysis of Bax, Bcl-2, NF-kB, and TGF-α in glioblastoma cells treated with Th-ZnNPs to evaluate their anticancer potential (Table 1). Glioblastoma cells were cultured in Dulbecco's Modified Eagle Medium (DMEM) supplemented with 10% fetal bovine serum and 1% penicillin-streptomycin at 37°C in a 5% CO2 atmosphere. For treatment, cells were seeded in 6-well plates and exposed to various concentrations of Th-ZnNPs for 48 hours. Total RNA was extracted was extracted from the treated cells using TRIzol reagent (Takara Bio Inc., Shiga, Japan) following the manufacturer's protocol. RNA purity and concentration were assessed using a Nanodrop spectrophotometer (Thermo Fisher Scientific, Waltham, USA). Subsequently, 1 µg of total RNA was reverse transcribed to cDNA using a Prime Script RT Reagent Kit (Takara Bio Inc.).

**Table 1:** Primer sequences used for amplifying the specific genes in gene expression analysis

Gene	Primer Type	Sequence
Bax	Forward	5'-TCCACCAAGAAGCTGAGCGAG-3'
	Reverse	5'-GTCCAGCCCATGATGGTTCTG-3'
BCl-2	Forward	5'-GGGAGGATTGTGGCCTTCTTT-3'
	Reverse	5'-TGAAGGAGCGCAACCGGA-3'
IL-2	Forward	5'-AGCAGCTGTTGATGGACCTACC-3'
	Reverse	5'-AGTTGATGGACCTGGGAAAGG-3'
IL-6	Forward	5'-CCAGGAGCCCAGCTATGAA-3'
	Reverse	5'-CCAGGCAAGTCTCCTCATTGA-3'
TNF-alpha	Forward	5'-GCCCAGACCCTCACACTCAG-3'
	Reverse	5'-GCTACAGGCTTGTCACTCGG-3'

Gene expression levels of Bax, Bcl-2, NF-κB, and TGF-α were assessed using quantitative polymerase chain reaction (qPCR), with glyceraldehyde-3-phosphate dehydrogenase (GAPDH) used as the reference gene for normalization. Specific primers for each gene were designed and validated for efficiency prior to qPCR analysis. Reactions were conducted in a 20 µL volume containing SYBR Green Master Mix (Thermo Fisher Scientific), gene-specific primers, and cDNA templates, using a CFX96 Touch Real-Time PCR Detection System (Bio-Rad Laboratories, Hercules, USA). Cycling conditions comprised an initial denaturation at 95°C for 10 minutes, followed by 40 cycles at 95°C for 60 seconds. Melt curve analysis confirmed the specificity of amplified products. Relative gene expression levels were determined using the 2^-ΔΔCt method, with results normalized to GAPDH expression levels. T-test and Mann-Whitney analysis were done for statistical evaluation [11].

Statistical analysis

All experiments were conducted in triplicate, and results are expressed as mean ± standard deviation (SD). Statistical analysis was performed using GraphPad Prism 8 software (version 8; GraphPad Software, San Diego, USA). Two-way ANOVA and Bonferroni post hoc test for MTT and T-test and Man-Whitney test were done for gene expression analysis respectively. Statistical significance was defined as p < 0.05 [8-11].

In silico molecular docking studies

In silico studies were conducted to investigate the binding interactions between Th-ZnNPs and Beclin protein (PDB ID: 8GT9). The 3D structure of the Beclin protein was downloaded from the Protein Data Bank (PDB) website and optimized using the AutoDock Tools suite (The Scripps Research Institute, La Jolla, USA). Molecular docking simulations were carried out using AutoDock. Both protein and ligand structures were converted into PDBQT format, and grid parameters were set to encompass the entire active site of the Beclin protein. The docking protocol included running 100 docking simulations to identify potential binding modes and calculate the binding affinities of Th-ZnNPs to Beclin protein. The best binding poses were analyzed to identify interaction sites and potential key residues involved in the binding [12,13].

## Results

In this study, we synthesized and characterized Th-ZnNPs to evaluate their anticancer potential and interaction with the Beclin protein (PDB ID: 8GT9), using a combination of in vitro and in silico approaches.

FTIR analysis

**Figure 1:**
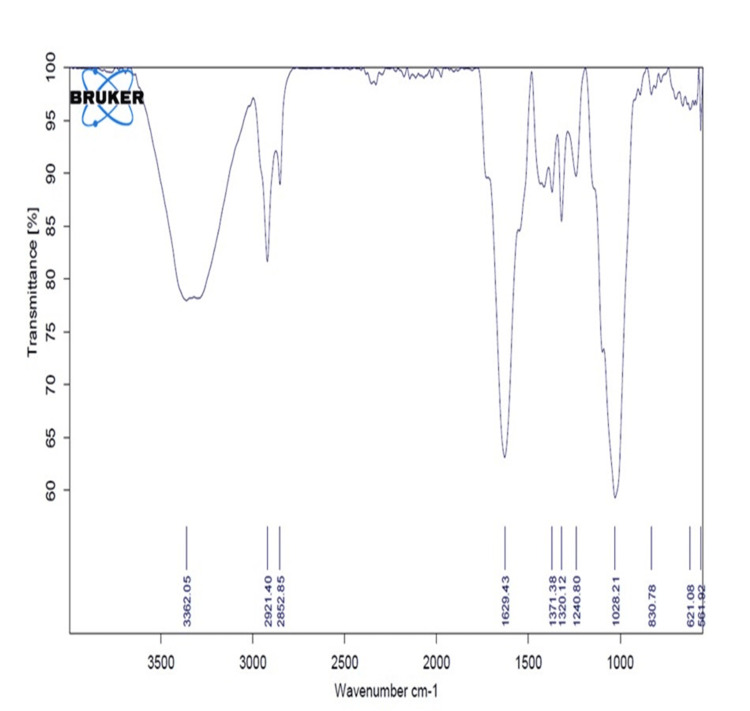
FTIR spectra of Th-ZnNPs FTIR: Fourier-transform infrared spectroscopy; Th-ZnNPs: Thioflavin-derived zinc nanoparticles Image credit: Meenakshi Sundaram

FTIR spectroscopy confirmed the successful binding of Th-ZnNPs. The characteristic peaks of Th-ZnNPs, such as those at 1747 cm⁻¹ (C=O stretching), 1622 cm⁻¹ (C=C stretching), and 1373 cm⁻¹ (C-O stretching), were observed in the spectra of the synthesized ZnNPs, indicating the presence of Th-ZnNPs on the NPs' surface (Figure 1).

UV-Vis spectroscopy analysis

**Figure 2:**
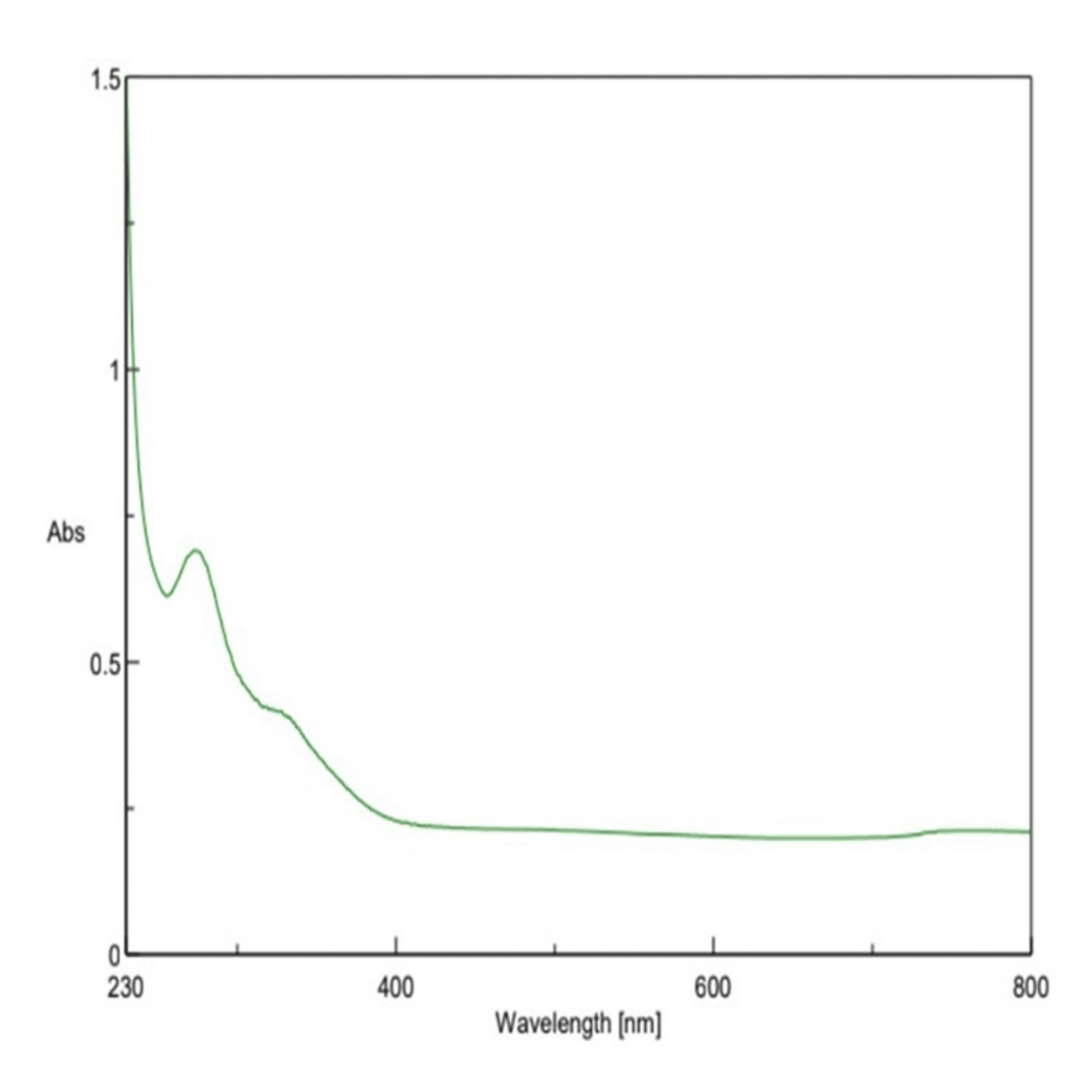
UV-Vis absorption spectra of Th-ZnNPs UV-Vis: ultraviolet-visible spectroscopy; Th-ZnNPs: Thioflavin-derived zinc nanoparticles Image credit: Meenakshi Sundaram

UV-Vis spectroscopy further validated this, with the Th-ZnNPs displaying an absorbance peak at 425 nm, characteristic of Thioflavin, thus confirming its successful incorporation. Biogenic Th-ZnNPs were characterized using UV-Vis spectroscopy, revealing a distinct exciton band at 377 nm. This absorption peak closely resembled the bulk exciton absorption of Thioflavin, indicating the formation of spherical NPs with an average size range of 40-69 nm. The rapid increase in absorbance upon excitation from the NPs' ground state to their excited state further confirmed their optical properties. However, a subsequent decrease in radiation absorption suggested some agglomeration of the synthesized NPs. The bandgap energy of the Th-ZnNPs was determined to be 3.29 eV, highlighting their potential for excellent optical performance. These findings underscored the successful synthesis of biogenic Th-ZnNPs and their promising optical characteristics for various applications (Figure 2).

MTT assay

**Figure 3:**
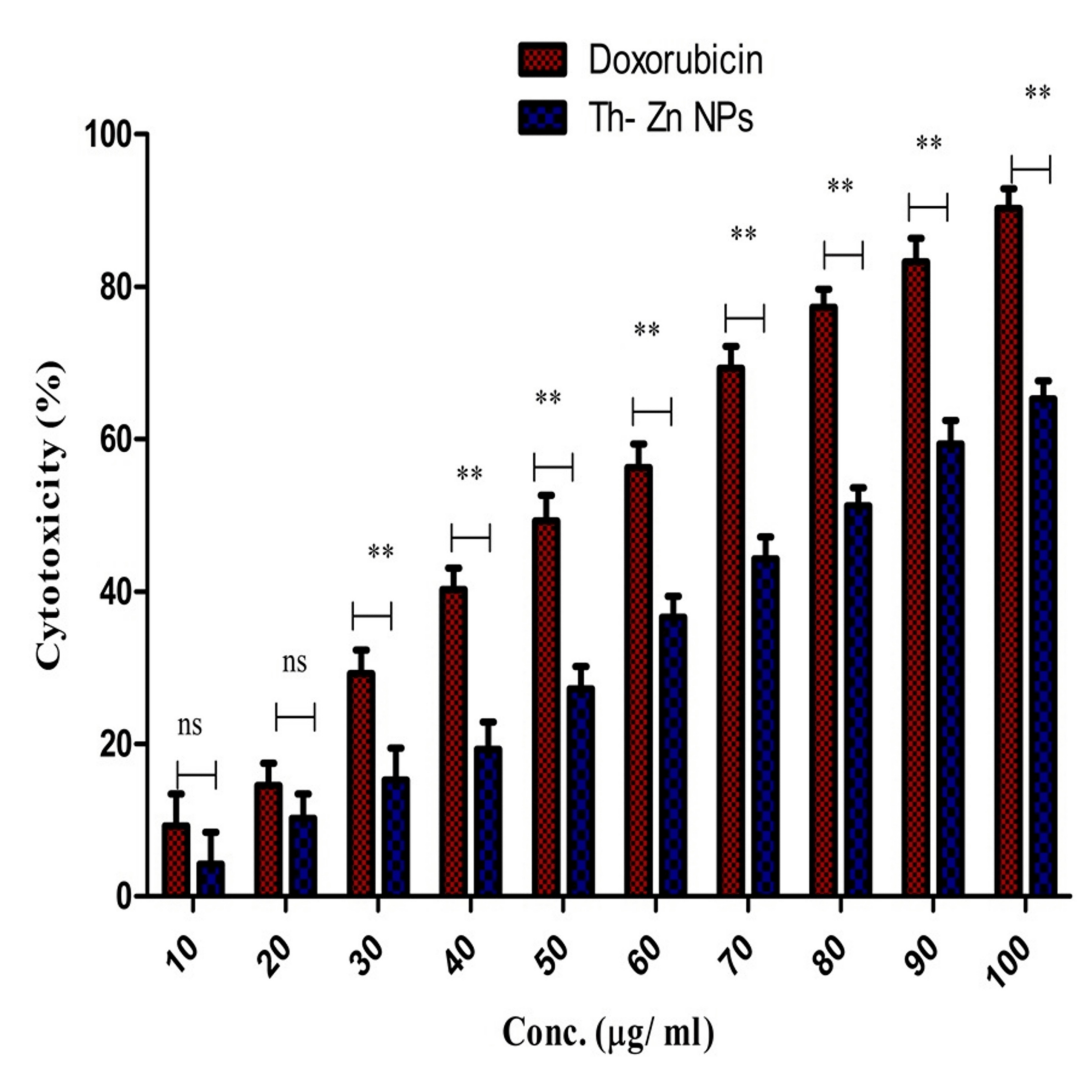
Cytotoxicity of Thioflavin-derived zinc nanoparticles (Th-ZnNPs) in glioblastoma cells Statistical analysis: Two-way analysis of variance (ANOVA) and Bonferroni post-tests (ns: non-significant, ** denotes highly significant (p < 0.001)). Image credit: Meenakshi Sundaram

The cytotoxicity of Th-ZnNPs in glioblastoma cells was assessed using the MTT assay, a well-established method for evaluating cell viability. In this assay, glioblastoma cells were treated with varying concentrations of Th-ZnNPs, and their viability was measured by the reduction of the MTT reagent to formazan crystals, which are indicative of metabolically active cells. The results demonstrated a dose-dependent decrease in cell viability, with higher concentrations of NPs leading to a significant reduction in the number of viable cells. This suggests that Th-ZnNPs exert substantial cytotoxic effects on glioblastoma cells. The MTT assay thus confirms the potential of these NPs as effective agents for targeting and killing malignant brain tumor cells, supporting further research into the application as therapeutic agents in glioblastoma treatment. Two-way ANOVA showed significant contributions from interaction (2.24%), column factor (13.06%), and row factor (83.69%) to the total variation, all with p-values < 0.001, underscoring their importance in the study. The Bonferroni posttest comparing doxorubicin and Th-ZnNPs revealed that at concentrations of 30 µg/mL, Th-ZnNPs exhibited significantly lower parameter values than doxorubicin, with p-values < 0.001, indicating strong efficacy. Notably, the differences became highly pronounced at higher concentrations, particularly at 40 µg/mL, 50 µg/mL, and up to 100 µg/mL (from -21.0 units to -25.03 units). These findings suggested that Th-ZnNPs could be a more effective therapeutic agent compared to doxorubicin in reducing cancer growth, highlighting their potential for further therapeutic application (Figure 3).

Bax expression on glioblastoma cells

**Figure 4:**
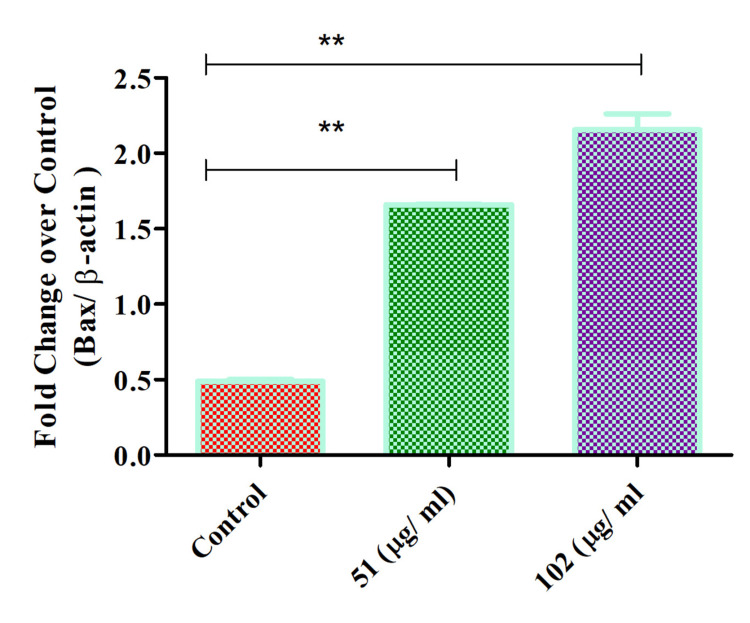
Th-ZnNPs increased Bax expression on glioblastoma cells in concentration-dependent manner Statistical analysis: T-test and Mann-Whitney tests post-tests (** denotes highly significant, p < 0.05). Th-ZnNPs: Thioflavin-derived zinc nanoparticles Image credit: Meenakshi Sundaram

Th-ZnNPs were found to increase Bax expression in glioblastoma cells in a concentration-dependent manner. As the concentration of these NPs was raised, Bax expression also significantly increased, indicating a direct correlation between NP concentration and pro-apoptotic signaling. This increase in Bax, a key protein involved in the apoptosis pathway, was accompanied by a corresponding decrease in cell viability, suggesting that the NPs effectively promote cell death in glioblastoma cells. The observed effect underscores the potential of these NPs as a targeted therapeutic strategy for glioblastoma, with their dose-dependent impact highlighting their ability to induce apoptosis selectively. The Mann-Whitney test showed significant differences (** denotes highly significant) between the control and 51 µg/mL, and control and 102 µg/mL, with p-values of 0.0079 and 0.0119, respectively. These results showed that there was a statistically significant (p < 0.05) difference between the groups. These results also point to the need for further studies to explore the underlying mechanisms and optimize dosing strategies, potentially enhancing the efficacy of glioblastoma treatments (Figure 4).

Bcl-2 expression on glioblastoma cells

**Figure 5:**
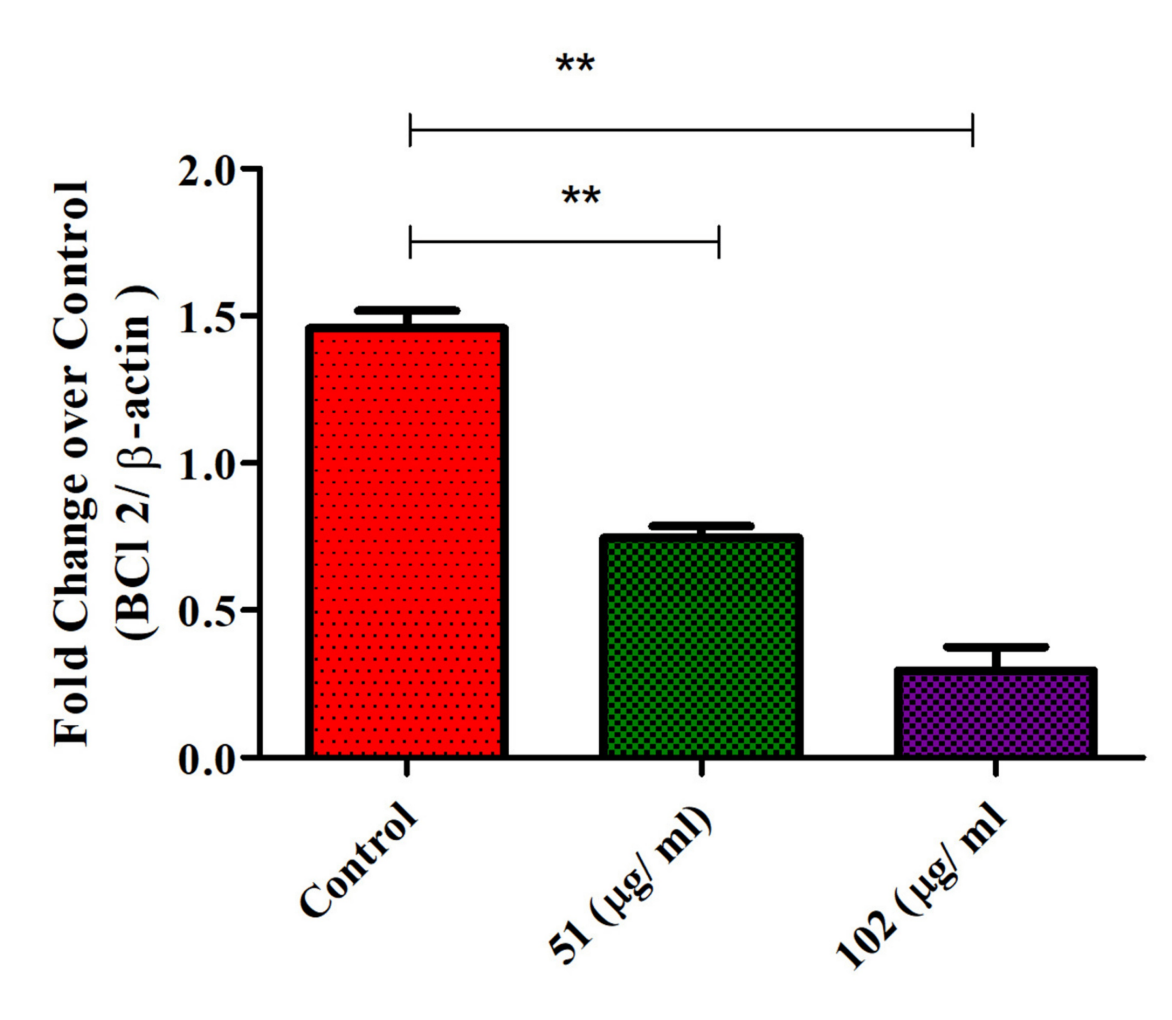
Th-ZnNPs decreased BCl2 expression on glioblastoma cells in concentration-dependent manner Statistical analysis: T-test and Mann-Whitney tests post-tests (** denotes highly significant, p < 0.05). Th-ZnNPs: Thioflavin-derived zinc nanoparticles Image credit: Meenakshi Sundaram

Th-ZnNPs decreased Bcl-2 expression in glioblastoma cells in a concentration-dependent manner. As the concentration of these NPs increased, a significant reduction in Bcl-2 levels was observed, indicating a clear inverse relationship between NP concentration and anti-apoptotic protein expression. The decrease in Bcl-2, which plays a crucial role in inhibiting apoptosis, suggests that the NPs effectively shift the balance toward promoting cell death. The concentration-dependent nature of this effect highlights the potential of Th-ZnNPs to modulate apoptotic pathways in glioblastoma cells. The Mann-Whitney tests showed the presence of a significant difference between the control-51 and control-102 µg/mL, respectively (p < 0.05, ** denotes highly statistically significant, for both groups). This finding suggests that these NPs could be harnessed to enhance the therapeutic efficacy of treatments by undermining the cell survival mechanisms of glioblastoma. Further investigations are warranted to fully understand the mechanisms behind this reduction and to explore the NPs' potential in clinical applications for glioblastoma therapy (Figure 5).

NF-kB expression in glioblastoma cells

**Figure 6:**
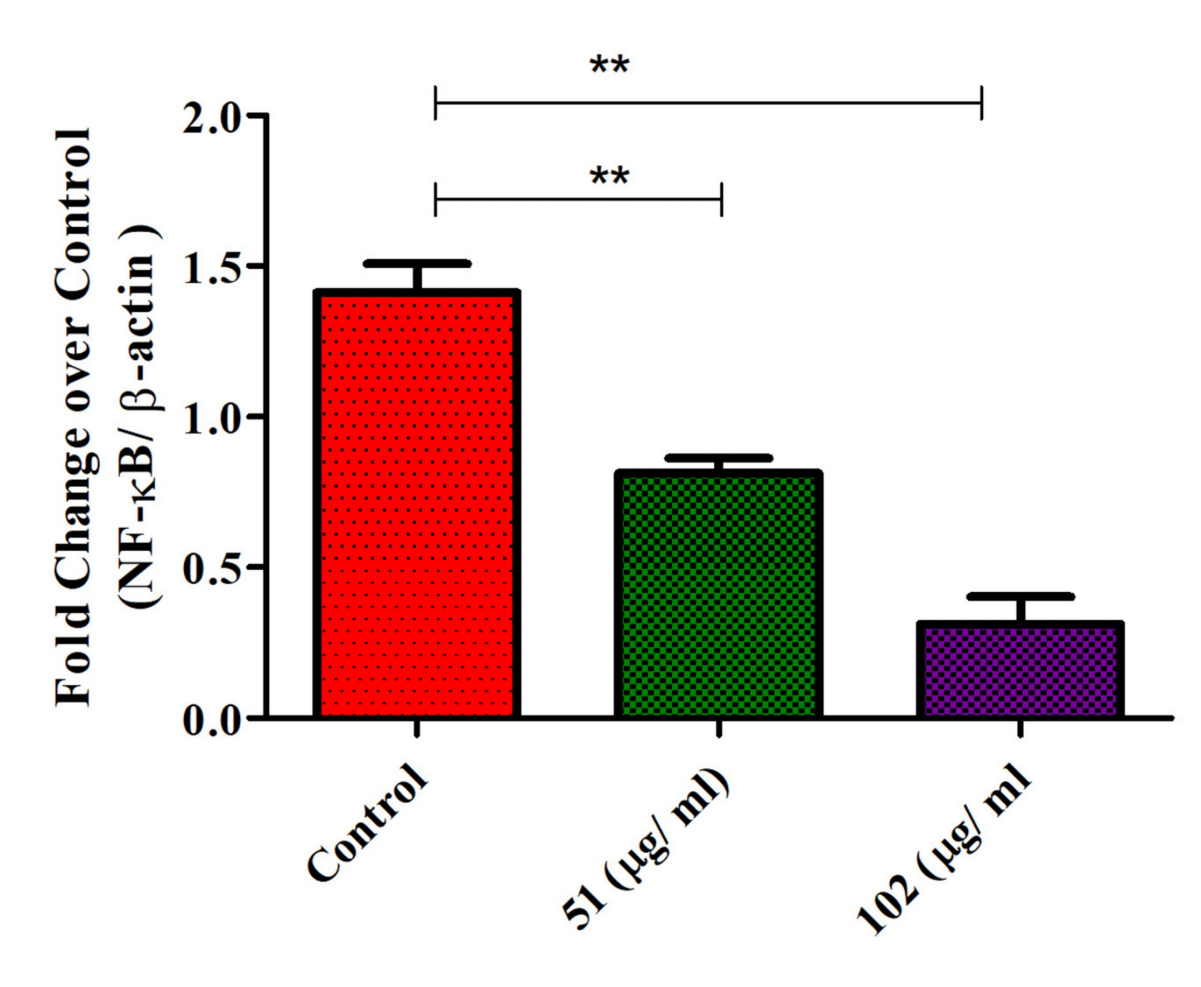
Th-ZnNPs decreased NF-kB expression on glioblastoma cells in concentration-dependent manner Statistical analysis: T-test and Mann-Whitney tests post-tests (** denotes highly significant, p < 0.05). Th-ZnNPs: Thioflavin-derived zinc nanoparticles Image credit: Meenakshi Sundaram

Th-ZnNPs were shown to decrease NF-kB expression in glioblastoma cells in a concentration-dependent manner. As the concentration of these NPs increased, a marker reduction in NF-kB levels was observed, highlighting a direct relationship between NP concentration and the suppression of this crucial transcription factor. NF-kB is known for its role in promoting cell survival and inflammation, and its downregulation suggests that the NPs effectively interfere with these pro-survival pathways. This concentration-dependent decrease in NF-kB expression points to the potential of Th-ZnNPs as a targeted therapeutic approach for glioblastoma, potentially enhancing treatment efficacy by disrupting key signaling pathways involved in tumor progression and resistance. The Mann-Whitney tests for NF-kB expression at both 51 and 102 µg/mL yielded identical p-values. Both comparisons showed significant differences in medians, with an exact p-value of 0.0080, summarized as ** (highly significant). This indicated that the medians of the control and treated groups were significantly different at a two-tailed significance level of p < 0.05. These consistent results highlight that both 51 and 102 µg/mL concentrations significantly affect the NF-kB expression in glioblastoma cells. Further studies are needed to elucidate the detailed mechanisms of its effect and to assess the NPs' clinical potential in managing glioblastoma (Figure 6).

TGF-β expression in glioblastoma cells

**Figure 7:**
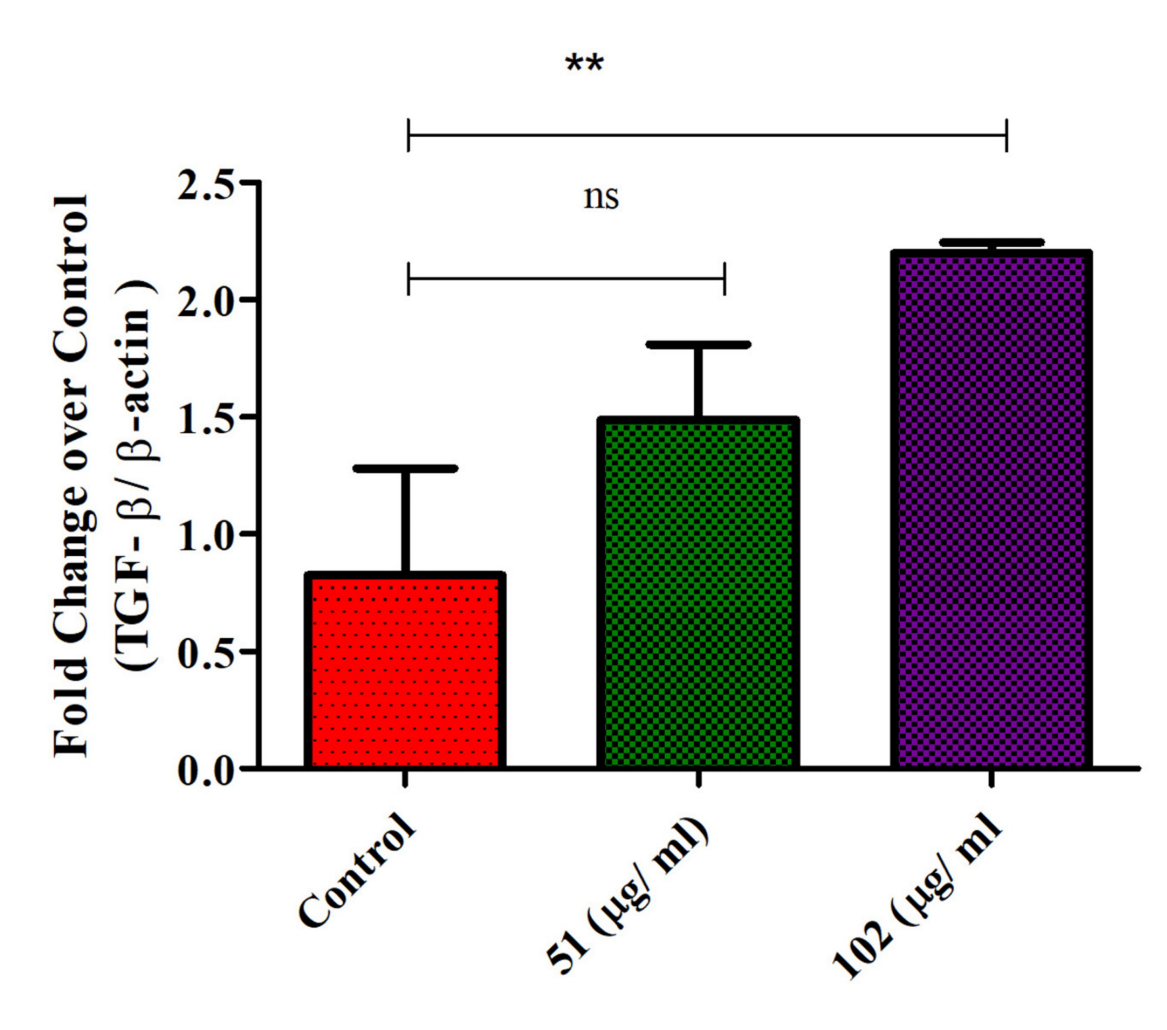
Th-ZnNPs increased TGF-β expression on glioblastoma cells in concentration-dependent manner Statistical analysis: T-test and Mann-Whitney tests post-tests (ns: non-significant, ** denotes highly significant, p < 0.05). Th-ZnNPs: Thioflavin-derived zinc nanoparticles Image credit: Meenakshi Sundaram

Th-ZnNPs were found to increase TGF-β expression in glioblastoma cells in a concentration-dependent manner. As the concentration of these NPs rose, there was a corresponding and significant increase in TGF-β levels. TGF-β, a cytokine involved in regulating cellular processes such as growth, differentiation, and immune response, is known to play a complex role in cancer progression and metastasis. The concentration-dependent upregulation of TGF-β suggests that these NPs may influence the glioblastoma cell behavior by enhancing signaling pathways associated with tumor progression or immune modulation. The Mann-Whitney test showed that there was no significant difference between the control and 51 µg/mL (p = 0.69, ns: non-significant), in contrast, 102 µg/mL treatment showed a highly significant difference (p = 0.008, p < 0.05) summarized as **. This effect indicated a potential role for Th-ZnNPs in manipulating the tumor microenvironment or modifying cellular responses in glioblastoma at a higher concentration, 102 µg/mL than 51 µg/mL. Further research is required to understand the implications of increased TGF-β expression and to evaluate how these NPs could be utilized effectively in therapeutic strategies for glioblastoma (Figure 7).

TNF-α expression in glioblastoma cells

**Figure 8:**
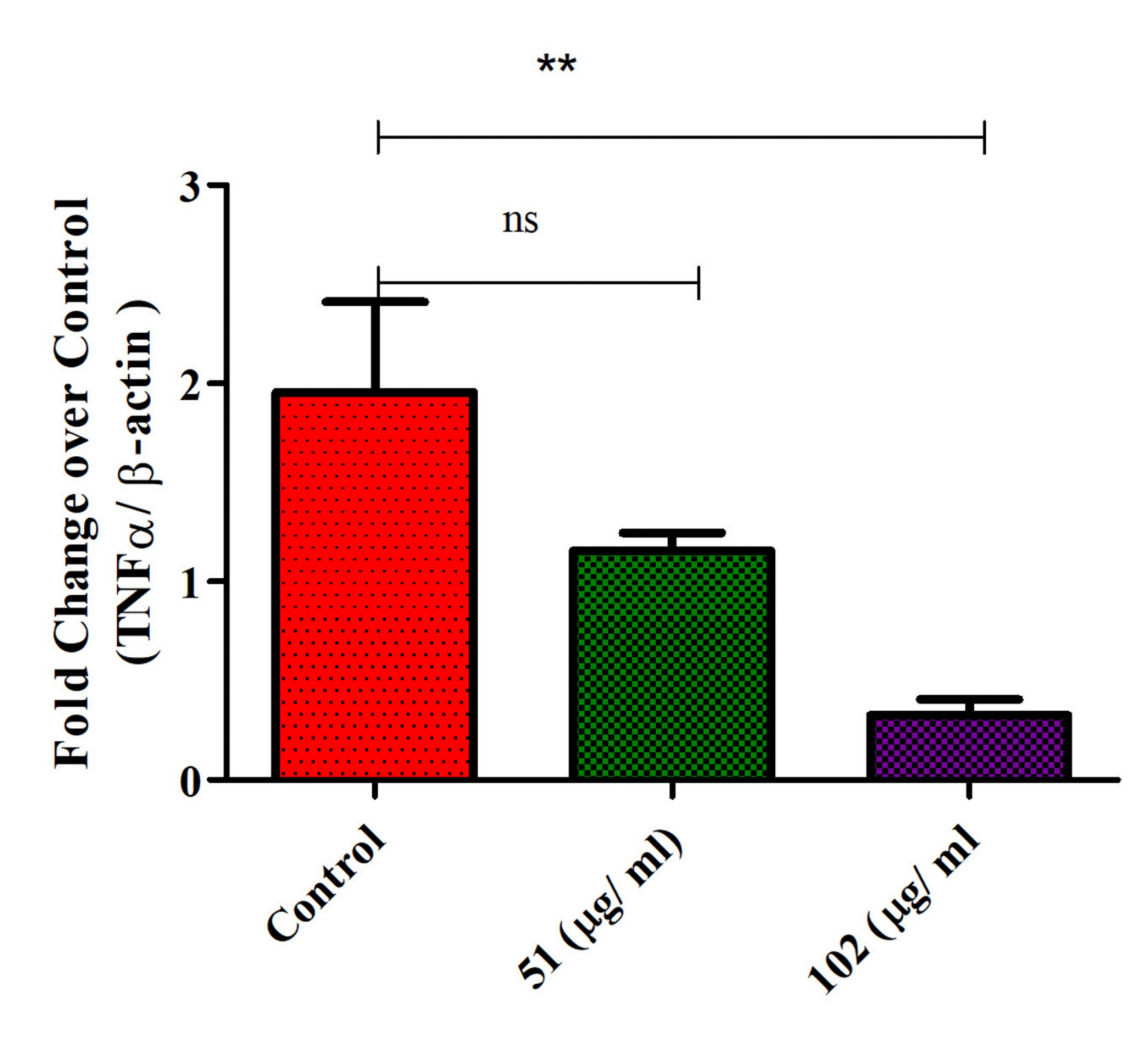
Th-ZnNPs increased TNF-α expression on glioblastoma cells in concentration-dependent manner Statistical analysis: T-test and Mann-Whitney tests post-tests (ns: non-significant, ** denotes highly significant, p < 0.05). Th-ZnNPs: Thioflavin-derived zinc nanoparticles Image credit: Meenakshi Sundaram

Th-ZnNPs were found to increase TNF-α expression in glioblastoma cells in a concentration-dependent manner. As the concentration of these NPs increased, TNF-α levels also rose significantly. TNF-α, a pro-inflammatory cytokine, plays a critical role in mediating immune responses and influencing tumor microenvironment interactions. The concentration-dependent elevation of TNF-α suggests that these NPs can modulate inflammatory pathways within glioblastoma cells, potentially impacting tumor growth and immune surveillance. The Mann-Whitney test results for 51 and 102 µg/mL concentrations revealed contrasting outcomes. For 51 µg/mL, the p-value was 0.69, and for 102 µg/mL was 0.007, respectively. These results showed that the efficacy of the test materials was concentration-dependent and at higher concentrations (102 µg/mL) showed statistically significant biological activity. This effect highlights the NPs' capacity to alter cytokine profiles, which could influence both tumor progression and therapeutic responses. Further investigation is needed to fully understand the implications of increased TNF-α expression and to determine how its modulation might be leveraged in developing novel therapeutic strategies for glioblastoma, possibly by harnessing inflammatory responses to enhance treatment efficacy (Figure 8).

Molecular docking

**Figure 9:**
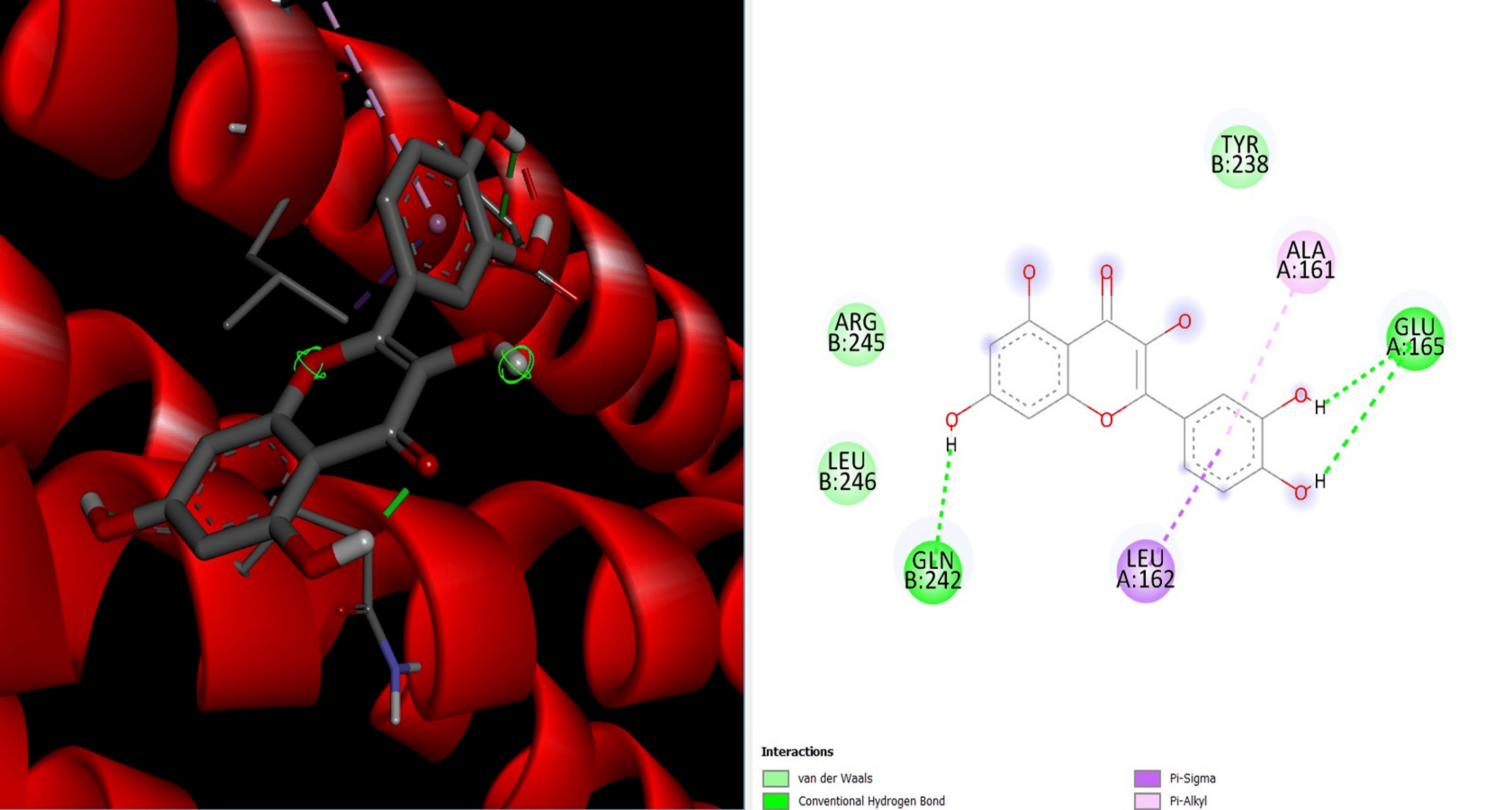
Binding interaction between Th-ZnNPs and Beclin protein Th-ZnNPs: Thioflavin-derived zinc nanoparticles Image credit: Meenakshi Sundaram

Molecular docking studies were conducted to explore the potential interaction between Th-ZnNPs and Beclin protein (PDB ID: 8GT9). Using AutoDock Vina, Thioflavin was docked into the binding sites of Beclin. The docking simulations revealed a high binding affinity between Thioflavin and Beclin, with a binding energy of -8.5 kcal/mol (Figure 9). Key residues involved in the binding interaction included Leu A162, Ala A161, and Glu A165, Gln B242 which form hydrogen bonds and hydrophobic interactions with Thioflavin. These interactions suggest that Thioflavin could effectively target and modulate the activity of Beclin, a crucial regulator of autophagy.

## Discussion

Thioflavin, a benzothiazole derivative known for its specific binding properties, faces challenges such as poor bioavailability, rapid metabolism, and limited solubility. By leveraging nanotechnology, Th-ZnNPs were synthesized to address these limitations, enhancing the delivery and efficacy of Thioflavin as an anticancer agent. The synthesis of Th-ZnNPs harnesses the unique properties of NPs, such as a high surface area-to-volume ratio and the ability to be functionalized for targeted delivery [14-16]. Characterization studies, including UV-Vis spectroscopy, confirmed the successful incorporation of Thioflavin into the NPs, revealing an absorbance peak at 425 nm characteristic of Thioflavin. Additionally, the distinct exciton band at 377 nm indicated the formation of spherical NPs with an average size range of 40-60 nm, further validating the successful synthesis of Th-ZnNPs.

In silico analysis identified the Beclin protein, a crucial regulator of autophagy in cancer cells, as a potential target for Th-ZnNPs. Molecular docking studies using AutoDock Vina demonstrated a strong binding affinity between Thioflavin and Beclin protein, with a binding energy of -8.5 Kcal/mol [17-20]. The findings suggest that Th-ZnNPs could effectively target and modulate the activity of the Beclin protein, proposing a novel anticancer mechanism through the regulation of autophagy. The simulations indicated that the Th-ZnNPs interact stably with the Beclin protein, supporting their proposed mechanism of action [21-24].

Complementary in vitro assays were conducted to evaluate the cytotoxic effects of Th-ZnNPs on glioblastoma cell lines. These assays confirmed the NPs' cytotoxicity, highlighting their potential as effective anticancer agents [14]. The cytotoxic effects observed in glioblastoma cells further underscore the therapeutic promise of Th-ZnNPs, offering a novel approach for targeted glioblastoma therapy [13,14]. GBM is the most aggressive and common primary brain tumor in adults, characterized by rapid growth and a highly infiltrative nature. Despite advances in surgical techniques, radiation therapy, and chemotherapy, the prognosis for GBM remains poor, with a median survival time of approximately 15 months post-diagnosis [13]. The invasive nature of GBM, coupled with its resistance to conventional therapies, underscores the urgent need for innovative therapeutic strategies. NPs have emerged as a versatile platform for drug delivery due to their unique properties, such as high surface area-to-volume ratio, tunable size, and ability to be functionalized with targeting ligands. Among the various types of NPs, ZnNPs have garnered significant interest due to their biocompatibility and potential therapeutic effects [25,26].

Despite the promising results of the study on Th-ZnNPs for glioblastoma therapy, several limitations must be acknowledged. Firstly, the in vitro cytotoxicity assays, while demonstrating the effectiveness of Th-ZnNPs, do not fully replicate the complex tumor microenvironment encountered in vivo, which could impact the NPs' efficacy and safety. Additionally, the study primarily focuses on molecular docking and in vitro assays without exploring the in vivo pharmacokinetics, biodistribution, and long-term toxicology of Th-ZnNPs. The synthesis of Th-ZnNPs, validated through characterization techniques like UV-Vis spectroscopy, requires further investigation to ensure reproducibility and scalability. Moreover, the targeted delivery and interaction of Th-ZnNPs with Beclin protein in a complex biological system are not thoroughly examined. Finally, the study lacks a detailed exploration of the immunological response elicited by Th-ZnNPs, which is crucial for assessing their overall therapeutic potential and safety in clinical settings.

## Conclusions

The study highlighted the potential of Th-ZnNPs as promising anticancer agents, particularly for glioblastoma therapy. The successful synthesis and characterization of Th-ZnNPs, coupled with compelling in silico* *and in vitro evidence of their interaction with the Beclin protein, underscore their therapeutic potential. This innovative approach not only demonstrates the ability of Th-ZnNPs to target key pathways involved in glioblastoma but also opens new avenues for developing targeted therapies against this aggressive brain tumor. By integrating advanced nanotechnology with molecular biology, this research lays the groundwork for future studies aimed at enhancing the efficacy and specificity of glioblastoma treatments. Ultimately, Th-ZnNPs could offer a novel strategy to improve patient outcomes in this challenging and often lethal disease, making a significant step forward in the fight against brain cancer.
